# Isolation, discrimination, and molecular detection of *Listeria* species from slaughtered cattle in Namwala District, Zambia

**DOI:** 10.1186/s12866-022-02570-6

**Published:** 2022-06-18

**Authors:** Prudence Mpundu, John Bwalya Muma, Nawa Mukumbuta, Andrew Nalishuwa Mukubesa, Walter Muleya, Penjaninge Kapila, Bernard Mudenda Hang’ombe, Musso Munyeme

**Affiliations:** 1 Department of Environmental and Occupational Health, Levy Mwanawasa Medical University, Lusaka, 33991 Zambia; 2 Department of Disease Control, School of Veterinary Medicine, University of Zambia, Lusaka, 10101 Zambia; 3 Department of Epidemiology and Biostatics, Levy Mwanawasa Medical University, Lusaka, 33991 Zambia; 4 Department of Biomedical Sciences, School of Veterinary Medicine, University of Zambia, Lusaka, 10101 Zambia; 5 Department of Para-Clinical Studies, School of Veterinary Medicine, University of Zambia, Lusaka, 10101 Zambia

**Keywords:** Beef carcasses, Contamination, *Listeria* species, Phenotypic

## Abstract

**Background:**

The food industry is increasingly becoming more scrutinized, given the frequency and intensity with which zoonotic diseases are being reported. Pathogen tracking has become more applicable with regards food safety. It is in this regard that the present study was formulated to track *Listeria* species. in freshly slaughtered cattle carcasses by utilizing standard and molecular biological techniques.

**Methods:**

A cross-sectional study design was conducted from March to December 2020 with 200 samples being equally collected in the rainy and dry seasons. A total of 180 and 20 swabs were aseptically collected from carcasses and the environment respectively. Samples were first subjected to pre-enrichment in half-strength Fraser broth followed by enrichment in full strength Fraser broth and subsequent plating on *Listeria* agar. *Listeria* growth characteristics were identified up to species level based on their morphological and biochemical characteristics. Further, molecular detection and phylogenetic analysis was conducted. Quantitative proportionate survey data were analyzed using Stata Version 15 software to estimate crude prevalence taking into account complex design at abattoir level. Factors associated with contamination were characterized using logistic regression. Sequences were analyzed using, Genetyyx version 12 and phylogenetic Mega.

**Results:**

Of the 200 samples, 19 were positive for *Listeria* species identified as *L.innocua* 14/19 (73.7%) and *L. monocytogenes* 5/19 (26.3%). All isolates were from freshly slaughtered carcasses, and none from environment. Siginificant differences in contamination levels were observed based on season: rainy season yielded 14 (73.6%) whilst the dry season 5 (26.3%). The *L. monocytogenes* strains showed a high degree of homogeneity on phylogenetic analysis and clustered based on abattoir. Seasonality was identified as a major determinant influencing contamination based on the final logistic regression model.

**Conclusion:**

This study found evidence of *L. monocytogenes* contamination on traditionally raised beef carcasses across various abattoirs surveyed. The failure to find *Listeria* contamination on the abattoir environment may to a greater extent intimate cattle carccases as primary sources of contamination. However, a more comprerehnsive study incorporating different geographical regions is needed to conclusively ascertain these present findings.

**Supplementary Information:**

The online version contains supplementary material available at 10.1186/s12866-022-02570-6.

## Introduction

Listeriosis infection is caused by *Listeria monocytogenes* (*L. monocytogenes*) bacteria of the genus *Listeria*. *L. monocytogenes* is a major pathogen that primarily affects pregnant women, newborns, older adults, and people with weakened immune systems [1]. People usually get infected with *Listeria* after eating contaminated food [2]. Cattle farms are an important array in spreading *Listeria* pathogens in food animals compared to small ruminant farms [3, 4]. The ruminant farm animals perpetuate the persistence of *Listeria* species in the rural environment via a continuous faecal-oral cycle [5, 6]. Moreover, the risk of Listeriosis in cattle increases when ensilage foods are provided or if animals graze on contaminated pasture [4, 7]. Although, other parameters such as good herd health management play a pivotal role in ensuring the microbiological quality of beef [8]. Listeriosis is an uncommon to cause of illness in the general population. The annual incidence of the European Union countries is 2–10 cases per million people [9].In the U.K., an outbreak of Listeriosis occurred, which affected pregnant women who purchased sandwiches from hospital-based retail shops [10]. A report in the United States indicates the incidence rates of 0.3 cases per 100,000 of Listeriosis in recent years transmitted via food [11]. Africa has a record of about 91 million people who have foodborne related diseases in 2015, while South Africa in 2019 recorded an outbreak of Listeriosis which was confirmed to come from a food source [12, 13]. While in Zambia, *Listeria* species, more specifically *L. monocytogenes* contamination, was detected in freshly cut organic vegetables sampled on farms grown for exportation [14]. Incidentally, *Listeria* species are reported to colonize a wide array of food products because of their ubiquitous nature in the environment [15]. The prevalence reports of *Listeria* species, including *L. monocytogenes,* in meat and raw meat products have been investigated in several countries [16, 17]. Additionally, *Listeria* species are post-processing contaminants that may arise due to inadequate cleaning and poor separation techniques between the ready to get foods and the raw foods [18].

Beef is among the known high-risk foods for pathogenic and non-pathogenic bacteria [16, 17]. Traditional meat inspections, lacking Good Manufacturing Practices (G.M.P.s), cannot assure the attainment and maintenance of high hygienic standards for meat regarding contamination with pathogenic bacteria such as *L. monocytogenes* [19]. The establishment of Hazard Analysis Critical Control Points (HACCP) is also important to ensure systematic control of meat slaughter processes regarding microbiological safety, spoilage, and storage stability [20]. Consequently, regulatory authorities are now moving towards the requirement for such systems in the meat industry [21]. Mostly, the traditional approach to assuring product quality involves inspections of sampled products from each batch and determining the proportion of samples that fail to meet the expected quality. Although this quality assurance method is usually feasible when throughputs are small, it becomes increasingly impractical as volumes increase [22].

The major contamination concerns in high-risk foods like beef are pathogens such as parasites, viruses, and most common bacteria [23]. Recently, several bacteria such as *Salmonella* and *Escherichia coli* that contaminate food origins have been documented in Zambia [24]. *Listeria,* more importantly, *L. monocytogenes,* is also among the group of bacteria of public health significance that is known to contaminate food. *Listeria* is ubiquitous in the environment, such as soil, manure, and grass [15]. *Listeria* species are facultatively anaerobic, non-spore-forming, a motile intracellular pathogen that comprises seventeen recognized species [25, 26]. Among the species of *Listeria,* the only one implicated in human infections is *L. monocytogenes* while *Listeria ivanovii* mainly affects ruminants [27]. More importantly is *L. monocytogenes* with a reported fatality of about 30%, while other species like *Listeria innocua (L.innocua)* and *Listeria seeligeri* are rarely pathogenic to humans. *Listeria,* by nature, maybe persistent in most food processing environments; once introduced in slaughter facilities may survive for years [28, 29]. Isolation of other *Listeria* species may indicate the absence of *L. monocytogenes* because it can be suppressed, especially in the presence of *L.innocua*; thus, this may increase the high rate of negativity for *L. monocytogenes* [30].

A study titled abattoirs, butcheries, and restaurants revealed high contamination in the dry season than wet season [31]. They attributed this difference to be because more samples were collected in the dry season than the wet season [32]. Other risks of *Listeria* contamination may be farm-specific such as the hygienic status of where the animals are kept, source of water, and feed [33]. In the same study, genetic relatedness of strains sampled from different farms was observed. The finding suggested, among others, carcass contamination originating from both incoming animals as well as transmission due to slaughter practices and persistent contamination coming from slaughterhouses [33].

The standard known microbiological methods routinely used for isolating *Listeria* species including *L. monocytogenes* in different samples usually require two enrichment steps (enriched with *Listeria* selective supplements) which are later inoculated on the surface of the *Listeria* selective agar [34]. The *prs* is a general marker gene present in all *Listeria* species, which encodes the enzyme phosphoribosyl pyrophosphate synthetase to determine the genus [35]. Other authors have also used *prs* to screen the presence of *Listeria* species because it is known as the housekeeping gene [35, 36]. In Zambia, molecular studies on *Listeria* are non-existent. It lacks information regarding the prevailing *Listeria* species; thus, this study incorporates culture and phylogenetic analysis to determine the prevalence, species, and strains of *Listeria* in the Namwala district of Zambia. Furthermore, the study aimed to determine the relationship and relatedness of the *Listeria* strains isolated in different seasons from different abattoirs in the Namwala district by analysing the *prs* gene.

## Methods

### Study design

A cross-sectional study design was conducted from March to December 2020 with 200 samples being equally collected in the rainy and dry seasons. The swabs were collected from the surfaces of carcass including cold room and storage environments in the abattoirs.

### Study site

Administratively, Zambia is divided into ten provinces, and among these provinces is the Southern province, which has the highest livestock-raising households accounting for 16%, with the majority residing in the Namwala district [37]. Namwala, which is located between the latitudes 15 and 170 South of the equator and longitude 25 and 270 East, has the greatest stretch of its traditional land covered by the Kafue flood plains, which offers nutritious varieties of green grass for wildlife, and approximately 300,000 cattle it houses [38]. It is also known to be the natural hub of traditionally reared beef produce supplied in most parts of Zambia [39]. As a result, several beef abattoirs are being constructed to answer the call of production [39]. Furthermore, most of the beef slaughtered in Namwala is not only consumed within but also supplied to all parts of Zambia [39].

Namwala District has six beef processing abattoirs [39], of which all were incorporated in this study except for one that was waiting to be commissioned for opening. The district was selected because it houses the largest number of abattoirs that supply beef on a small scale and commercial bases throughout the country [38]. Furthermore, Southern Province is reported to contribute the highest number of cattle compared to other Provinces [37]. The abattoirs involved were identified as one, two, three, four, and five.

#### Sample size and sampling

One hundred eighty carcass swabs and 20 environmental swabs were collected from five beef abattoirs. Sample size estimatation was based on an assumed prevalence of 27.5% [40] at 80% power and a 5% significance level. The two hundred samples were divided equally as one hundred each for exterior and interior carcass swabs, including sampling season. All swabs were collected immediately after evisceration and hide removal. The total maximum throughput for all the abattoirs for a day was reported to be 150 carcasses, the stated sample size was equally allocated to the abattoirs, and a total of 36 carcass swabs and four environmental samples per site were collected. The abattoirs, having the same maximum capacity throughput, slaughtered an average of 30 carcasses per day, with only one recording slaughters of 90 per day as a maximum. Complex design was employed to account for bias brought about by oversampling and under-sampling of certain abattoirs. Simple random sampling was the technique that was used to pick the carcass for swabbing through shuffling before the next pick was done.

#### Sample collection and processing

Bacteriological standard sample collection for *Listeria* contamination was used on samples collected from five abattoirs [41]. A template metal that was sterile was used to outline 5 × 5cm^2^ area parts marked for swabbing, including environmental swabs [42]. Surface swabs were collected from the interior and exterior parts of the carcasses. The outlined areas by the metal template were swabbed with a sterile moist cotton gauze which was wrapped around the end of a flat swab stick. Swab samples were placed in screw-cap tubes containing Amies transport media [41]. The swab samples were identified according to date, ingredient samples (e.g. Beef carcass), batch code, and site name, including comments specific to the sample (e.g. interior or exterior), were recorded. All samples were given codes for easy identification according to sampling site/product or ingredient type, date, and site further, and these samples were all kept at -4ºC before being transported within 72 h at the Microbiology laboratory at the School of the Veterinary Medicine University of Zambia. The samples were immediately transferred in 9 ml of pre-enrichment broth and later incubated at 37 ºC for 48 h. Before isolation from swab diluents, samples were vortexed for 30 s and then plated on selective media to detect the target micro-organisms [42] in both environmental and carcass swab samples.

#### Isolation and identification of *Listeria* species

The beef carcass swabs were tested for the presence of *Listeria* species using Standard international methods which were recommended by the International Organization for Standardization (ISO g11290 -1: 1996, 2004) procedure. First, a 1 g of the sample representative portion from each was inoculated in 9 ml of pre-enriched broth and incubated at 37 ºC for 24 h, then 1 ml of pre-enriched broth was transferred into 9 ml of Fraser broth (Oxoid) (enriched with *Listeria* selective supplement) and vortexed for 1 min, followed by incubation at 37 ºC for 48 h. A loop-full of pre-enrichment broth (Oxoid) culture was inoculated on the surface of *Listeria* selective agar (Oxoid), incubated at 37 ºC for 48 h, and observed for colonies showing growth typical greenish sheen morphology or green–blue colonies' colour of *Listeria*. The suspected colonies were then sub-cultured onto Nutrient Agar (Oxoid) and later incubated at 37 ºC for 24 h to obtain pure colonies. Some standard biochemical tests were done on the purified cultures, namely, Gram's staining, citrate, urea, indole, motility, oxidase, catalase, and methyl-red tests to obtain a presumptive diagnosis of *Listeria*.

#### DNA extraction and PCR Identification of *Listeria*

Following biochemical tests, polymerase chain reaction (PCR) assays were performed to confirm the presumptive isolates of *Listeria* species. D.N.A. was extracted from the pure culture of the suspected isolates, grown on nutrient agar using a commercial genomic D.N.A. extraction kit (ZYMO Research quick D.N.A. miniprep kit) as per the manufacturer's instructions. The primer pairs designated as *prs* -F (5'- GCT GAA GAG ATT GCG AAA GAA G – 3') and *prs*-R (5’-CAA AGA AAC CTT GGA TTT GCG G- 3') were used to amplify a 370 bp fragment of the *Listeria prs* gene [43]. A 50 µl PCR master mixture, consisting of 5 ul of 10 × PCR buffer, 1.5 ul of 0.5 ul of Taq D.N.A. polymerase, 1 ul of 10 mM dNTP5 mix (10 Mm l µl), 100 ng of template and Nuclease. The thermal cycler conditions were: initial denaturation at 94 ºC for 2 min followed by 35 cycles, denaturation at 94 ºC for 45 s, annealing at 53 ºC for 45 s, and extension at 72 ºC for 2 min with a final extension, at 72 ºC for 7 min. The amplified PCR products were visualized on 1% agarose gel coated with ethidium bromide.

#### Purification of PCR products and cycle sequencing

The amplified PCR products were purified using a Promega purification kit (Wizard S.V. Gel & PCR Promega clean-up System) per the manufacturer's instructions [44]. According to the manufacturer's instructions, the Purified PCR products were then subjected to sequencing PCR reaction using brilliant dye terminator ver.3.1 kits according to the manufacturer's instructions. The sequence products were precipitated as described by [45] after denaturation with formamide were then subjected to capillary electrophoresis on the ABI 3500 Genetic Analyzer [46].

#### Data analysis

The obtained data from the beef carcasses and the storage environment was entered in the Excel sheet and imported to Stata version 15 (Stata cop, college station, Texas, U.S.A.) for all analyses. The primary outcome was *Listeria* or *Listeria* species (i.e. *L. innocua* and *L. monocytogenes*) contamination from various beef carcass swabs, including environmental swabs. The prevalence of *Listeria* contamination was calculated as the proportion of the total beef carcasses collected that were contaminated, taking into account proportional weights for abattoir throughputs. Factors associated with *Listeria* contamination such as seasons, part swabbed, and abattoir name were investigated, considering complex design at abattoir level (clusters). To account for confounders, forward stepwise model building was used by conducting univariate logistic regression, and all factors that were significant inclusion in the multivariable logistic model was considered. Complex design was used to account for intra –cluster correlation in this study. All analyses in this study were stratified by site, and a *p*-value of 0.05 was used to determine statistical significance using a likelihood ratio test.

#### Sequence analysis

Nucleotide sequences obtained in this study were first subjected to blast analysis on the NCBI website (https://blast.ncbi.nlm.nih.gov/Blast.cgi) to verify the species of bacteria or *Listeria* obtained, followed by assembly and editing using the ATGC plug-in Genetyx ver. 12. Using the obtained sequences and reference sequences downloaded from the GenBank, a multiple sequence alignment was constructed using clustalW1.6 (Supplementary Fig. 1). Furthermore, a fasta file of the multiple alignments was generated using MEGA 6 [47, 48] and utilized to construct a neighbour-joining phylogenetic tree with 1000 bootstrap replicates as a measure of the confidence interval [47, 48]. All the generated sequences in this study have been deposited in the DNA Data Base of Japan with accession numbers LC629080 to LC629098 (Supplementary Table 1).

## Results

### Descriptors of *Listeria* prevalence

Of the 200 environmental and beef carcasses collected,19 had *Listeria* species of which 20 came from environmental swabs and 180 from carcass swabs. When specific *Listeria* contamination in the abattoirs was considered, *L. innocua* accounted for 14 (73.7%) and *L. monocytogenes* 5 (26.3%).

Furthermore, when specific establishment contamination was considered abattoir one, showed the highest contamination of both *L. innocua* 8 (42.1%) and *L. monocytogenes* 4 (21.1%). Meanwhile, no *Listeria* species were isolated from the environmental samples collected in this study. (Table 1).

**Table 1:** Prevalence of isolated *Listeria* species in beef carcasses (*n* = 19)

Variable	Prevalence of *L. innocua* (%)	Prevalence *of L. monocytogenes* (%)	Prevalence of* Listeria* species (%)
Abattoir			
1	8 (42.1)	4 (21.1)	12 (63.2)
2	2 (10.5)	1 (5.3)	3 (15.8)
3	1 (5.3)	0 (0.0)	1 (5.3)
4	0 (0.0)	0 (0.0)	0 (0.0)
5	3 (15.8)	0 (0.0)	3 (15.8)
Totals	14 (73.7)	5 (26.3)	19 (100)

### Phylogenetic analysis

**Fig. 1:**
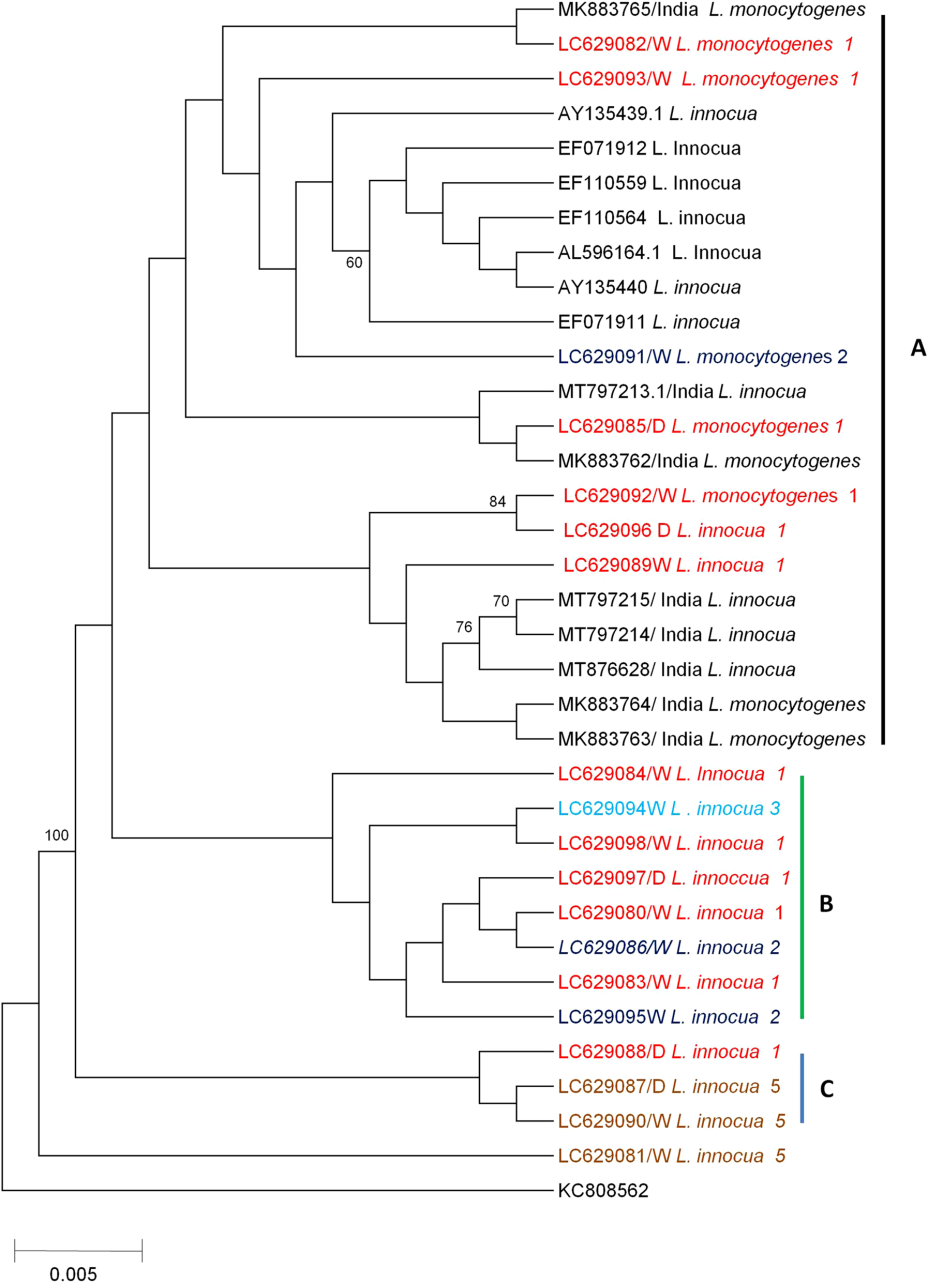
Phylogenetic tree. Comprising of 35 sequences (19 obtained in this study and 16 downloaded sequences from (GenBank) based on 370 bp partial nucleotide sequences of the *prs* gene of *Listeria*. The tree was inferred using the Neighbor-joining method with 1000 bootstrap replicates as a confidence interval. The Maximum Composite likelihood method was used to compute the evolutionary distances with all ambiguous positions for each sequence pair removed. The color codes and number at the end of the study sequences represent the abattoir of origin and the season of sampling is designated as D; dry season and W; wet season

Phylogenetic analysis of the obtained sequences as well as the reference sequences showed the presence of three clusters, namely clusters A, B and C (Fig. 1). Cluster A comprised of both *L. monocytogenes* and *L.innocua* reference sequences as well as study sequences collected in both the dry and wet season from abattoir 1 and 2, with the majority originating from abattoir 1. On the other hand, clusters B and C exclusively comprised of *L. innocua* isolated from this study. In cluster B, sequences from abattoir 1, 2 and 3 collected both during the dry and wet season formed a cluster while in cluster C, sequences from abattoir 1 and 5 were present. Sequence LC629081 from this study did not cluster in any of the above clusters, however it was closely related with sequences from C (Fig. 1). Overall, phylogenetic analysis revealed that sequences collected in both the dry and wet season from abattoir 1 were represented in all clusters, while sequences from abattoir 5 were only present in cluster C. Clustering according to seasonality was not observed.

Univariate analysis of *Listeria* species and *L.innocua* indicated season of sampling to be significantly related to contamination level (*p* < 0.0032) and (*p* < 0.0101), respectively; (Tables 2 and 3).

**Table 2:** Univariate analysis showing *Listeria* contamination according to abattoir, season variation and sampling site (*n* = 200)

Variable	*Listeria* contamination		*P*-value
	**Contaminated (%)**	**Not contaminated (%)**	
Abattoir			
1	12 (6.0)	28 (14.0)	0.1952
2	3 (1.5)	37 (18.5)	
3	1 (0.5)	39 (19.5)	
4	0 (0.0)	40 (20.0)	
5	3 (1.5)	37 (18.5)	
Total	19 (9.5)	181 (90.5)	
**Season Variation**			
Dry	5 (2.5)	95 (47.5)	0.0032
Wet	14 (7.0)	86 (43.0)	
Total	19 (9.5)	181.0 (90.5)	
**Sampling Site**			
External	8 (4.0)	92 (46)	0.4596
Internal	11 (5.5)	89 (44.5)	
Total	19 (9.5)	181 (90.5)

**Table 3:** Univariate analysis showing *L. innocua* contamination according to abattoir, season variation and sampling site (*n* = 200)

Variable	*L. innocua*		*P*-value
	**Contaminated (%)**	**Not-contaminated (%)**	
Abattoir			
1	8 (4.0)	32 (16.0)	0.2612
2	2 (1.0)	38 (19.0)	
3	1 (0.5)	39 (19.5)	
4	0 (0.0)	40 (20.0)	
5	3 (1.5)	37 (18.5)	
Total	14 (7.0)	186 (93.0)	
**Seasonal variation**			
Dry	3 (1.5)	97 (48.5)	0.0101
Wet	10 (5.0)	90 (45.0)	
Total	13 (6.5)	187 (93.5)	
**Sampling Site**			
External	6 (3.0)	94 (47.0)	0.8574
Internal	8 (4.0)	92 (46.0)	
Total	14 (7.0)	186 (93.0)	

A logistic regression analysis was carried out to determine the strength of the association of factors that were likely to influence *Listeria* contamination in abattoirs. The season in which the sampling was done was significant for *Listeria*, with samples collected during the wet seasons having (OR = 3.31; 95% CI: 1.27–5.35) odds of contamination compared to the dry season. Similarly, the parts swabbed were equally significant, with the internal parts having (OR -1.17; 95% CI: -2.49 -1.59) odds of contamination compared to the external parts (Table 4).

**Table 4:** Factors associated with *Listeria* contamination

Variables	Unadjusted			Adjusted		
**Season Variation**						
Dry	^a^**(ref)**			^a^**(ref)**		
Wet	7.40	0.005	2.71– 20.20	3.31	0.014	1.27 – 5.35
Abattoir						
1	^a^**(ref)**			^a^**(ref)**		
2	1.94	0.001	1.94 – 1.94	–1.75	0.001	(–2.06) – (–1.44)
3	0.59	0.001	0.59 – 0.59	–2.99	0.001	(–3.32) – (–2.65)
4	1	-	-	0	-	-
5	1.89	0.001	1.89 – 1.89	–1.91	0.001	(–2.30 – (–1.51)
**Sampling Site**						
External	^a^**(ref)**			^a^**(ref)**		
Internal	1.65	0.463	2.97 – 9.17	–1.17	0.068	(–2.49) – (1.59)

^a^ (ref) means "represents the reference category when interpreting the OR"

A logistic regression analysis was used to determine the strength of the association of factors that were likely to influence *L*. *innocua* contamination in abattoirs. The season in which the sampling was done was found significant for *L. innocua*, with samples collected during the wet seasons having (OR 24.59; 95% CI: 1.64 – 368.8.7) odds of contamination compared to the dry season. Similarly, the parts swabbed were equally significant, with the internal parts having (OR 0.12; 95% CI: 0.09 – 1.67) odds of contamination compared to the external parts (Table 5).

**Table 5:** Factors associated with *L. innocua* contamination

Variables	Unadjusted			Adjusted		
	**OR**	***P*-value**	**95%**	**OR**	***P*-value**	**95% CI**
**Season Variation**						
Dry	^a^**(ref)**			^a^**(ref)**		
Wet	4.59	0.013	1.69 – 12.42	24.59	0.033	(1.64) – (368.7)
Abattoir						
1	^a^**(ref)**			^a^**(ref)**		
2	2.16	0.001	(2.16) – (2.16)	3.00	0.008	(1.64) – (5.51)
3	1.03	0.001	(1.03) – (1.03)	1.39	0.002	(0.74) – (2.63)
4	1	-	-	1	-	-
5	3.24	0.001	(3.24) – (3.24)	4.12	0.025	(2.09) – (8.12)
**Sampling Site**						
External	^a^**(ref)**			^a^**(ref)**		
Internal	8.57	0.858	0.91 – 8.06	0.12	0.083	(0.09) – (1.67)

^a^ (ref) means "represents the reference category when interpreting the OR"

## Discussions

To the best of our knowledge, this study is the first in Zambia to isolate, determine and characterize *Listeria* from traditionally raised cattle carcasses from abattoirs. The unique ability of *Listeria* to survive food preservation or hostile environments, coupled with its long incubation period, makes it a serious threat to food safety and may potentially result in it being missed by diagnosticians and clinicians. In this study, we utilized molecular phylogenetic analysis to determine the relatedness of the isolated *Listeria* species and determine the proportion of different strains of *Listeria*. From this analysis, we identified two species of *Listeri*a based on culture, biochemical tests, and finally, through gene sequencing of *prs* as *L. monocytogenes* and *L. innocua*. These present findings of 26.3% as *L. monocytogenes* is slightly higher than what was reported by Nguz et al. in (2005) who reported a prevalence of 20% in freshly cut vegetables harbouring *L. monocytogenes* [14]. Despite that their study was in vegetables, compared to this present study in meat, their findings presented a much lower percentage prevalence variance. Apart from the difference in sample sources, to some extent, the inconsistency in prevalence may conservatively be attributed to differences in the identification methods used between the two studies; Nguz et al. (2005) only utilized differential and selective agars (PALCAM and OXFORD agars) without any definitive molecular methods while in the current study, molecular techniques were utilized through PCR and sequencing of *prs* gene.

Molecular techniques are more reliable and have high differentiation power within and between organisms that exhibit similar characteristics compared to cultural methods [49]. To this regard, the present study attempted to further confirm the identity of *Listeria* species using PCR and sequencing of the *prs* gene. Blast analysis of the obtained sequences showed a similarity score ranging from 89% to 99.9% (Supplementary Table 1) and phylogenetic analysis revealed clustering of sequences under study (mainly from abattoir 1) with *L. monocytogenes* and *L. innocua* reference sequences in cluster A while other sequences under study formed exclusive clusters B and C (Fig. 1). Thus, despite the *prs* gene not being entirely able to disciminate *Listeria* to species level [43], through PCR and sequencing of the *prs* gene coupled with culture and biochemical tests, this study was able to ascertain the different types of *Listeria* species as *L. innocua* and *L. monocytogenes* based on Blast analysis (https://blast.ncbi.nlm.nih.gov/Blast.cgi) and the pattern of clustering on phylogenetic analysis (Fig. 1) [49]. One of the sequences (LC629092), despite showing a similarity score of 98.75% with *L. monocytogenes*, closely clustered with *L. innocua* in cluster A (Fig. 1). This observation can be attributed to the biological relatedness that exist between *L. monocytogenes* and *L.innocua* [50]. Furthermore, sequences from different abattoirs clustered together irrespective of the sample origin or season (Fig. 1) implying that the abattoir could be the source of contamination and not necessarily the farms because *Listeria* contamination was linked only to specific abattoirs with others ( i.e. abattoir 4) recording zero contamination. In addition, all sequences from abattoir 5 clustered together, further ascertaining the notion that the abattoir was the primary source of contamination. The data presented in this study is thus in agreement with previous studies were *L. monocytogenes* was observed to be endemic in specific abattoirs [51, 52].

In the recent past, *L. monocytogenes* caused a major outbreak in other African countries like South Africa [13], Zambia’s major trading partner, especially regarding the food of both animal and plant origin. During the same outbreak, the isolation of *L. monocytogenes* was reported mostly in cold meats, i.e. polonies [13]. Wieizorek's and others reported 19.4% of *L. monocytogenes* in beef meat samples [53]; in Malaysia, *L. monocytogenes* in meat samples was 8.6% [16], in Poland, bovine carcasses were found positive with *L. monocytogenes* at 2.5% attribution. While, in Iran, a lower prevalence was detected with only a single sample found contaminated with *L. monocytogenes* 2.7% [54], in the same country R.T.E. food samples were found contaminated with *L. monocytogenes* 19.1% [50]. This contamination difference can be linked to the handling processes that the R.T.E. foods undergo compared to the raw beef.

Furthermore, the above prevalence disparities recorded in other studies compared to the results of this current study can mainly be ascribed to methodological differences of isolation of the micro-organism in question [49]. The other important aspect can be aligned to the differences in the sampled foods and the ability of *Listeria* to survive in the same foods [55]. More importantly, *Listeria* is an ubiquitous bacteria in the environment and it is mainly introduced in the food due to inadequate hygienic practices were stringent measures are not employed this can also bring about differences in prevalence contamination in different studies reported [56].

This study also showed that *L. innocua* was the major contaminant.This is an important finding, because other studies have long suggested its usefulness as an indicator of the presence of *L. monocytogenes* [30]. Mostly *L. innocua*, although non-pathogenic to humans, may indicate lapses in food control systems in processing abattoirs. Mainly, contaminating bacteria are associated with the absence of prerequisite programs that help in ensuring food safety quality. In this present study, most contaminating bacteria were isolated in abattoirs that were observed to lack proper food management systems such as the implementation of Standard Operating Systems (SOSs) including HACCP. HACCP is a scientific tool that helps in the identification of hazards systematically [20, 27]. *L. monocytogenes* and *L. innocua* are common species in food processing plants, with the latter being prevalent; therefore, continuous monitoring is needed to avoid there existence [30, 57]. Therefore, when investigating the sources of *L. monocytogenes*, the isolation of *L.innocua* is equally high because it is commonly known to colonize food premises [30]. *L. innocua* has been reported by others to be more prevalent in food processing environments than *L. monocytogenes* [58]. Although the adaptive nature of *L.innocua* in the food processing environment is not fully understood, this is helpful information in controlling food pathogens [58]. Additionally, further studies are needed to ascertain the presence of *L. monocytogenes* and *L. innocua* if they are influenced by the respective environment (i.e. farms, food processing facilities, or foods). This could indicate whether one or more species is more likely to persist through the farm to fork continuum.

Meanwhile, no *Listeria* species were isolated from environmental swabs collected from storage cold-rooms in this study. The sample size allocated to the environmental swabs could have partly influenced the none isolation of *Listeria* species as only 20 swabs were collected from each abattoir. The other factor could be linked to the short storage of carcasses in the studied abattoirs after slaughter. Normally the carcasses from these abattoirs are only kept in the cold rooms for a maximum of two days and are later dispatched to their final destination. This, to a greater extent could have facilitated adequate cleaning, which could have been prevented if carcasses were stocked in the abattoir longer [59].

Contrary to this result, the finding in another study reported having isolated *Listeria* species on environmental samples 54.7% [60]. These variances in isolation can probably be explained by differences in the sampled environment concerning the storage system of carcasses and the length of storage from the two studies. The other differences can also be drawn from the hygienic conditions of specific abattoir facilities because *Listeria* is known to form biofilms that are resistant to most disinfectants commonly used in processing abattoirs [61].

From the total average number of contamination, the majority were recorded in the wet and dry seasons. *Listeria* is ubiquitous in the environments like soil, manure, and grass; this is comparable to free-range grazing of pasture in the dry season, which reduces the chances of cross-contamination because the feeding is off-site where animals are sheltered [15]. Other reports concluded the same with this current study with the highest number of *L. monocytogenes* recorded in the rainy season 3%, while dry 0.8% [62]. The rainy season in most parts of Zambia is cooler compared to the dry, hot season when the sampling took place. The characteristic nature of *Listeria* it thrives in cold environments and foods kept at extended refrigeration conditions [63]. There is some consistency in the above findings about contamination levels of this current study with regards to the season of sampling, and this can be attributed to the ubiquitous nature of *Listeria* and its ability to withstand the cold weather environment. During the wet season, its survival ability could have been enhanced due to moisture and cool temperatures.

Additionally, during grazing, the animals are most likely to consume viable *Listeria* pathogens available in the pasture because of the conducive environment provided in the wet season. Bacteria multiplication, among others, depends on moisture availability; this could partly explain the differences in the isolation rates in this given study [64]. This current study gives a snapshot of *Listeria* prevalence from sampling in the dry and wet season mouths at commercial abattoirs, and differences not found in the report may be attributable to several factors, including processing plants, the weather, and more importantly sources of cattle presented for slaughter on the sampled days.

The *L. monocytogenes* species, especially in raw beef carcasses, poses public health threat mainly when the meat is consumed raw or undercooked. More importantly, the other risk can be through cross-contamination during production at retail levels, especially in processing areas with poor hygienic practices. Incidences of Listeriosis mostly cumulates from consuming contaminated food items like R.T.E. foods, sea foods, dairy, vegetables, and beef carcasses [50, 65]. Therefore, it is important to ensure the safety of the natural products because the quality of the final product largely depends on it, as earlier indicated in the study done in poultry abattoirs [22]. Changing consumer trends such as the consumption of raw vegetables and undercooked foods such as beef are major reasons for causing foodborne infections [66]. Danger is created, especially if beef is contaminated with pathogenic micro-organisms such as *L. monocytogenes* [67]. Codex Alimentarius Commission, an international regulating body, has set guidelines on the allowable limits of *L. monocytogenes* in different types of foods to help producers easily perform quality checks on their food products [68].

Contamination variances of *Listeria* contamination were recorded across the abattoirs in this study, with some recording more to zero contamination. Mainly contamination differences are expected where there are environmental dissimilarities such as slaughter throughputs schedules of individual abattoirs, with some having more than others. Increased workload may have an influence on the frequency of cleaning especially in hard to reach cervices. Observation was done in a study of poultry abattoirs where increased contamination was recorded in an abattoir with high process throughput [22]. Further evidence was seen from the results reported in another study of abattoirs which displayed a similar picture of the current results of this survey [69]. Abattoir designs, especially those without clear separation between the clean and the dirty section, pose a huge risk of contamination on the food product compared to those abattoirs [22, 69]. As earlier elaborated, differences may also arise due to lack of hygienic practices by the food handlers working in the processing abattoirs, quality of water used, including environmental factors of the farms where these animals are coming from [70, 71].

The part-swabbed was also found significantly related to the contamination of *Listeria* species, with the internal part being more contaminated than the outer swabbed part. Contrary to this current findings, results were reported indicating no significant *Listeria* contamination with the parts swabbed [72]. Discrepancies recorded in the two studies could also be linked to the beef samples collected by Eruteya and colleagues who sampled cut pieces at the market.Uniform distribution may represent bacteria across all parts of the beef meat, unlike the carcasses sampled from the abattoirs. Some portions like external parts were not exposed to cutting compared to internal parts. The linkage is attributable to the handling and use of the knives or equipment that may be unsterilized from one carcass to another.High possibility of intestinal contaminants may aid the spreading through the internal part. The beef carcasses slaughtered in the investigated abattoirs were sourced from various farms with unknown risk parameters that we may not explain due to lack of supporting data. In abattoirs where adherence to good hygienic practices lacks the possibility of cross-contamination may occur through various utensils used. Other studies have elaborated the role of knives used for evisceration and cutting of carcasses to be among the major drivers of cross-contamination [71, 73, 74].

## Conclusion

This study has been able to elucidate the presence of *L. monocytogenes* and *innocua* in traditionally raised beef carcasses across various abattoirs in the Namwala district of Zambia using traditional and molecular methods. Additionally, in the present study*, L. innocua* was isolated at a comparatively higher rate than *L. monocytogenes.* The failure to find *Listeria* contamination in the abattoir environment may, to a greater extent intimate cattle carccases as primary sources of contamination. However, a more comprerehnsive study incorporating different geographical regions and increased sample size will be needed to affirmatively and conclusively ascertain our present findings.

## Supplementary Information

**Additional file 1:** **Supplementary Figure 1.** Multiple sequence alignment of downloaded reference sequences and sequences generated in this study. Multiple sequence alignment was performed using ClustalW1.6.

**Additional file 2: Supplementary Table 1.** Description of samples collected from cattle in Namwala district in 2020.

## Data Availability

All sequences emanating from this study have been deposited in the D.N.A. Data Base of Japan with accession numbers LC629080 to LC629098. All the data concerning this manscript are contained within the text.

## Abbreviations

ATGC: Adenine, Thymine, Guanine, Cytosine
HACCP: Hazard Analysis Critical Point
WHO: World Health Organization
G.M.P.s: Good Manufacturing Practices
D.N.A.: Deoxyribonucleic acid
PCR: Polymerase Chain Reaction
rRNA: Ribosomal ribonucleic acid
prs: Phosphoribosyl pyrophosphate synthetase
C.S.O.: Central Statistical Office
N.C.C.: Namwala Municipal Council
e.g.: Example given
ISO: International Standard Organization
g: Gram
cm^2^: Centimeters squared
^0^ C: Degrees Celsius
NCBI: National Center for Biotechnology Information
MEGA6: Molecular Evolutionary Genetics Analysis version 6.0
ACEIDHA: Africa Center of Excellence for Infectious Diseases of Humans and Animals
MFLCSO: Ministry of Fisheries and Livestock Central Statistical Office
ml: Millie litre
ppm: Parts per million
CI: Confidence Interval
SOSs: Standard Operating Systems
