# Supplementary figures and images for: Isolation, discrimination, and molecular detection of Listeria species from slaughtered cattle in Namwala District, Zambia
Source: BMC Microbiol. 2022 Jun 18;22:160. doi: 10.1186/s12866-022-02570-6 (PMC9206240; doi:10.1186/s12866-022-02570-6)

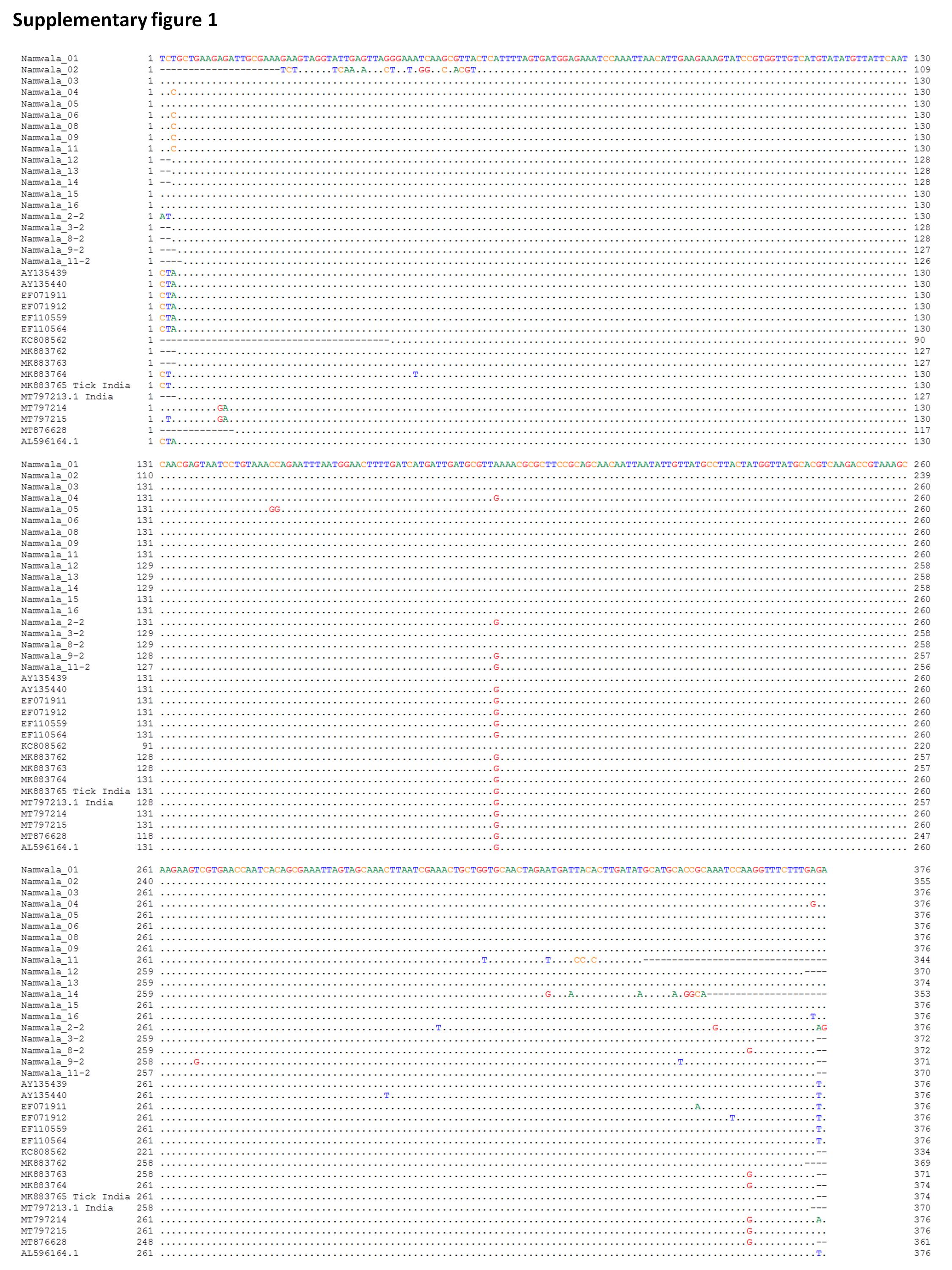

Supplement: Supplementary file 1 — Additional file 1: Supplementary Figure 1. Multiple sequence alignment of downloaded reference sequences and sequences generated in this study. Multiple sequence alignment was performed using ClustalW1.6. [file 12866_2022_2570_MOESM1_ESM.jpg]
