# Supplementary material for: Isolation, discrimination, and molecular detection of Listeria species from slaughtered cattle in Namwala District, Zambia
Source: BMC Microbiol. 2022 Jun 18;22:160. doi: 10.1186/s12866-022-02570-6 (PMC9206240; doi:10.1186/s12866-022-02570-6)
Supplement: Supplementary file 2 — Additional file 2: Supplementary Table 1. Description of samples collected from cattle in Namwala district in 2020. [file 12866_2022_2570_MOESM2_ESM.docx]

**Supplementary Table 1.** Description of samples collected from cattle in Namwala district in 2020

| **Sample** | **Site of collection/swabbing** | **Abattoir** | **Accession number** | **Blast analysis similarity score (%)** | **Reference accession number** |
| --- | --- | --- | --- | --- | --- |
| Namwala_01 | Internal carcass | 01 | LC629080 | 93.15 | CPO45743.1 |
| Namwala_02 | External carcass | 05 | LC629081 | 99.38 | KC808562.1 |
| Namwala_2-2 | Internal carcass | 01 | LC629082 | 99.37 | MK883765.1 |
| Namwala_03 | Internal carcass | 01 | LC629083 | 98.74 | CP045743.1 |
| Namwala_3-2 | External carcass | 01 | LC629084 | 98.48 | CP054846. 1 |
| Namwala_04 | External carcass | 02 | LC629085 | 95.30 | KC808562.1 |
| Namwala_05 | Internal carcass | 02 | LC629086 | 99.38 | KC805562.1 |
| Namwala_06 | External carcass | 05 | LC629087 | 99.9 | KC808562.1 |
| Namwala_08 | Internal carcass | 01 | LC629088 | 98.75 | CP045743.1 |
| Namwala_8-2 | Internal carcass | 01 | LC629089 | 99.38 | KC808562.1 |
| Namwala_09 | Internal carcass | 05 | LC629090 | 99.38 | KC808562.1 |
| Namwala_9-2 | External carcass | 01 | LC629091 | 98.79 | MK883765.1 |
| Namwala_11 | External carcass | 01 | LC629092 | 97.86 | MK8837763 |
| Namwala_11-2 | Internal carcass | 01 | LC629093 | 92.98 | CP054846.1 |
| Namwala_12 | Internal carcass | 03 | LC629094 | 89.00 | CP045743.1 |
| Namwala_13 | Internal carcass | 02 | LC629095 | 99.38 | KC808562.1 |
| Namwala_14 | External carcass | 01 | LC629096 | 98.75 | CPO45743.1 |
| Namwala_15 | Internal carcass | 01 | LC629097 | 99.38 | KC808562.1 |
| Namwala_16 | External carcass | 01 | LC629098 | 99.9 | KC808562.1 |
